# Bone Lining Cells Could Be Sources of Bone Marrow Adipocytes

**DOI:** 10.3389/fendo.2021.766254

**Published:** 2021-12-03

**Authors:** Ji Yeon Lee, Jae-Yeon Yang, Sang Wan Kim

**Affiliations:** ^1^Department of Internal Medicine, Seoul National University College of Medicine, Seoul, South Korea; ^2^Department of Research and Experiment, Seoul National University Hospital Biomedical Research Institute, Seoul, South Korea; ^3^Division of Endocrinology and Metabolism, Seoul Metropolitan Government Boramae Medical Center, Seoul, South Korea

**Keywords:** bone marrow adipocyte, bone lining cell, anti-sclerostin antibody, rosiglitazone, osteoblast

## Abstract

**Background:**

Recently, lineage-tracing studies demonstrated that parathyroid hormone and anti-sclerostin antibody (Scl-Ab) can convert bone lining cells (BLCs) into active osteoblasts. However, BLCs might also be differentiated into other lineages. Here we investigated whether BLCs could differentiate into bone marrow adipocytes (BMAds) and whether Scl-Ab could suppress this process.

**Methods:**

Dmp1-CreERt2:mTmG mice were injected with 0.5 mg of 4-hydroxytamoxifen once weekly from postnatal week 4 to week 8. The mice were treated with either vehicle or rosiglitazone for 8 weeks (weeks 12–20). Moreover, they were administered either vehicle or Scl-Ab (50 mg/kg) twice weekly for 4 weeks (weeks 16–20, N = 4–6/group). We chased the GFP+ cells from the endosteal surface to the bone marrow (BM) of the femur. Using immunohistochemical staining, the numbers of perilipin+ or GFP+/perilipin double+ cells in the BM were quantified. In addition, serum N-terminal propeptide of type I procollagen (P1NP) levels were measured at each time point, and bone mass was analyzed at 20 weeks using micro-computed tomography.

**Results:**

Scl-Ab administration significantly reversed the decreases in bone parameters induced by rosiglitazone. Plump GFP+ cells, presumably active osteoblasts, and extremely flat GFP+ cells, presumably BLCs, were present on the endosteal surface of the femur at 8 and 12 weeks, respectively, in line with prior findings. When we chased the GFP+ cells, rosiglitazone significantly increased the number of GFP/perilipin double+ BMAds compared to the effects of the vehicle (P < 0.001), and overlapping Scl-Ab administration decreased the number of GFP/perilipin double + BMAd compared to rosiglitazone alone (P < 0.001). In addition, we found that osteoblast lineage cells such as BLCs might express PPARγ on immunohistochemical staining. When rosiglitazone was administered to Rip-Cre:mTmG mice, GFP+ cells were not present on the endosteal surface or in the BM of the femur; however, they were present in the pancreas.

**Conclusion:**

BLCs could be sources of BMAds, and rosiglitazone could stimulate the differentiation of osteoblast lineage cells into BMAds. Suppression of the differentiation of osteoblast lineage cells into BMAds might contribute to anabolic effects resulting from the pharmacologic inhibition of sclerostin.

## Introduction

Bone marrow adiposity (BMA) is a specific fat depot in bone cavities. BMA increases with age, and it is caused by a variety of induction signals including thiazolidinediones, glucocorticoids, high-fat diet feeding, and irradiation exposure (1). Under these conditions, bone marrow adipose tissue (BMAT) could replace hematopoietic/osteogenic marrow in the long bones. A large body of research has revealed an inverse relationship between BMA and bone mineral density (BMD) in young or elderly men and women (2–5). In addition, postmenopausal women can exhibit the most consistent association compared to old men (2, 3). Furthermore, vertebral fractures were associated with a higher BMA volume in women with postmenopausal osteoporosis, and BMA was also associated with measures of decreased bone integrity (6, 7). Interestingly, 1 year of teriparatide treatment resulted in decreased vertebral BMAT with concomitant increases in lumbar spine BMD in postmenopausal women (8).

Bone marrow adipocytes (BMAds) are distinct from white or beige adipocytes in terms of localization, function, and origin (9–11). Previous lineage-tracing studies demonstrated that BMAds do not share the same progenitors as extramedullary adipocytes, and they might be derived from bone marrow (BM) (12, 13). The definite origin of BMAds remains unclear. Recent studies revealed that BMAds are derived from skeletal stem cells (SSCs) in BM, and Osx+, LepR+, and Nes+ SSC populations are capable of generating BMAds (14–16). Thus, the origin of BMAds might be heterogeneous.

Bone lining cells (BLCs) are quiescent osteoblasts covering bone surfaces. BLCs are sources of active osteoblasts and target cells for anabolic agents. Short-term treatment with parathyroid hormone (PTH) or anti-sclerostin antibody (Scl-Ab) can induce the conversion of BLCs into active osteoblasts (17, 18). In addition, BLCs express stem cell-like genetic markers (19). Those studies suggested that BLCs have the potential to differentiate into other lineages (20). Thus, we investigated whether BLCs could represent one source of BMAds. In addition, we examined whether Scl-Ab administration could suppress the possible transdifferentiation of BLCs into BMAds. To better understand the response of BLCs to adipogenic signals and follow their subsequent differentiation, we conducted a lineage-tracing study using inducible transgenic mice.

## Materials and Methods

### Mice

Temporally controlled transgene expression was used to trace cells of the osteoblast lineage using Dmp1-CreERt2 and mTmG mice. We used the mouse 10-kb Dmp1 promoter to drive the expression of the inducible CreERt2 in transgenic mice because it is expressed not only in osteocytes but also in mature osteoblast cell populations. The mutated ERt domain responds only to the synthetic estrogen receptor ligand tamoxifen. Administration of tamoxifen induces transient nuclear translocation and CreERt-mediated gene recombination. Dmp1-CreERt2 mice were crossed with mTmG mice, a double fluorescent reporter mouse strain. A reporter gene, such as the Rosa26R mTmG reporter transgene, can visualize the recombination since the expression of cre-recombinase induces the permanent excision of the upstream cassette encoding the membrane-targeted dTomato (mT) reporter protein to allow expression of a downstream cassette encoding a membrane targeted eGFP (mG) reporter protein (21, 22). In this manner, cells expressing cre-recombinase are indicative of mG expression. Then, mG is expressed continuously in the target cells and all possible progeny. Therefore, in this system, we can chase the fate of GFP-expressing cells such as from osteoblast lineage cells to BMAds (12). The 10-kb Dmp1-CreERt2 mice the Rip-Cre mice were kindly provided by Dr. Henry Kronenberg (Endocrine unit, Massachusetts General Hospital, USA) and Dr. Hye Seung Jung (Seoul National University Hospital). The 10-kb Dmp1-CreERt2 mice were generated by nuclear injections of a transgene encoding CreERt2 under the control of a 10-kb fragment of Dmp1 in B6C3F1 hybrid mice (Taconic, Hudson, NY, USA). Reporter mice (*Gt(ROSA)26Sortm4(ACTB-tdTomato,-EGFP)Luo/J*, stock no. 007576) were purchased from The Jackson Laboratory (Bar Harbor, Maine, USA).

These studies were approved by the Institutional Animal Care and Use Committee of Seoul National University.

### Tamoxifen Administration

For experiments with Dmp1-CreERt2:mTmG mice, 4-hydroxytamoxifen (4-OHTam, Takeda, Osaka, Japan) was used. For 4-OHTam injections, 2.5 mg of 4-OHTam were dissolved in 100 mL of dimethylformamide (Thermo Fisher Scientific, Waltham, MA, USA) and then diluted to 2.5 mg/mL in corn oil (Sigma-Aldrich, St. Louis, MO, USA). Dmp1-CreERt2 were injected with 0.5 mg of 4-OHTam once weekly in postnatal weeks 4–8.

### Rosiglitazone and Sclerostin Antibody (Scl-Ab) Administration

**Figure 1:**
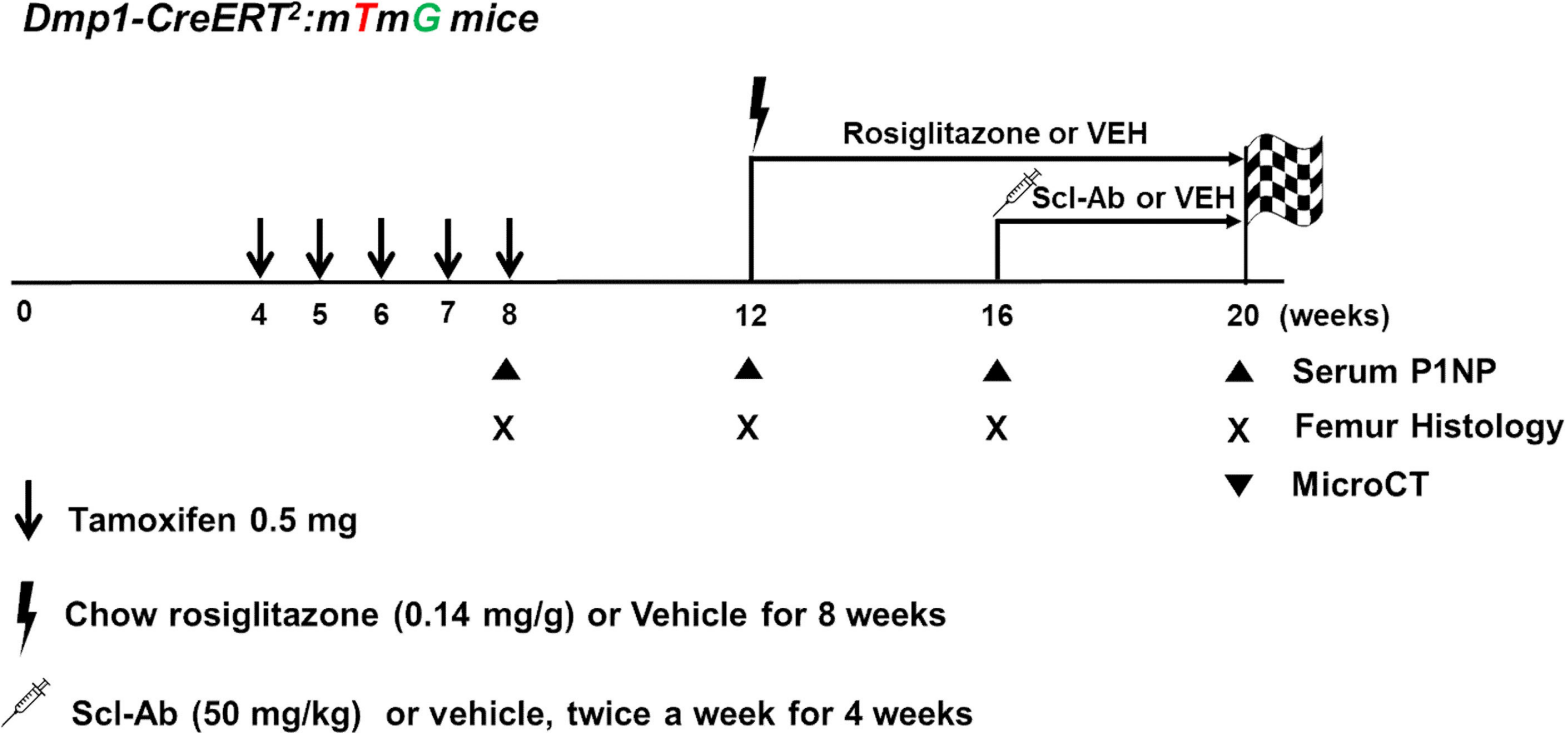
Experimental design. Dmp1-CreERt2:mTmG mice were injected with 0.5 mg 4-OHTam on postnatal weeks 4, 5, 6, 7, and 8. The mice were treated with either vehicle or rosiglitazone for 8 weeks (weeks 12–20). Moreover, they were administered either vehicle or Scl-Ab (50 mg/kg) twice weekly for 4 weeks (weeks 16–20, N = 4–6/group). We chased the GFP+ cells from the endosteal surface to the bone marrow of the femur. Animals were euthanized on postnatal week 8 or 12 (2 days or 4 weeks after the last 4-OHTam treatment), as well as on week 16 and 20 to evaluate the impact of rosiglitazone or Scl-Ab administration.

In postnatal week 12, the mice were fed a 20 mg/kg/day rosiglitazone-supplemented diet (containing 140 mg rosiglitazone/kg diet; R0106, Tokyo Chemical Industry, Japan) or chow diet ad libitum for 8 weeks (randomly assorted into cages and group-housed), weighed, humanely euthanized, and analyzed. In addition, mice were injected subcutaneously with vehicle (phosphate-buffered saline [PBS]) or 50 mg/kg Scl-Ab (ratized antibody; Amgen Inc., Thousand Oaks, CA, USA; UCB, Brussels, Belgium) twice weekly for 4 weeks starting in postnatal week 16 (**Figure 1**). Mice were sacrificed in postnatal weeks 8 (+2 days), 12, 16, and 20.

### Serum Biochemistry

Blood was collected *via* orbital sinus puncture before animals were euthanized. The serum levels of N-terminal propeptide of type I procollagen (P1NP) were measured using an ELISA kit (Immunodiagnostic Systems, Fountain Hills, AZ, USA).

### Micro-Computed Tomography (micro-CT)

Whole femurs were harvested from the mice after euthanasia, fixed in formaldehyde solution for 48 h, placed in 70% ethanol, and stored at 4°C until imaging. The distal femur from each mouse was scanned using high-resolution micro-CT (SkyScan 1173, Bruker microCT, Kontich, Belgium) at 90 kV and 88 μA with an isotropic voxel size of 7.1 μm using a 1.0-mm aluminum filter. For the metaphyseal tibia, a 1.5-mm section (starting 500 μm below the growth plate) was analyzed. Scanned images were reconstructed using NRecon v.1.6 software (Bruker microCT) by correcting for beam hardening and ring artifacts. Data were analyzed using CT Analyzer version1.6 (Bruker microCT). For trabecular bone regions, bone volume density (BV/TV), trabecular thickness (Tb.Th), trabecular separation (Tb.Sp), and trabecular number (Tb.N) were assessed.

### Histology and Immunohistochemical Staining

Samples were dissected to remove soft tissues, fixed in 4% paraformaldehyde, incubated overnight at 4°C, and then decalcified in 15% EDTA for 7–14 days. Decalcified samples were cryoprotected in 30% sucrose/PBS followed by a 30% sucrose/PBS : OCT (1:1) solution, each performed overnight at 4°C. Samples were then embedded in OCT and cryosectioned at 8 μm (Leica, CM3050S). Images were captured using an epifluorescence microscope (Leica DMI 6000 [Inverted]) with prefigured triple-band filter settings for DAPI/FITC/TRITC and an automated fluorescent microscope with a whole-slide scanning platform. Confocal images were acquired using LEICA TCS SP8 STED CW and LAS X (Leica) software with lasers and corresponding band-pass filters for DAPI (Ex. 405 nm, BP 420–480), GFP (Ex. 488 nm, BP 505–530), and tomato (Ex. 543 nm, BP 565–595). Representative images of at least three independent biological samples are presented in the figures. Routine H&E staining was performed using previously published protocols.

Paraffin sections were postfixed in 4% paraformaldehyde for 10 min, blocked with 2% BSA for 30 min, and incubated with mouse anti-GFP monoclonal antibody (1:250, ab6673, Abcam), and anti-perilipin antibody (1:250, ab3526, Abcam) overnight at 4°C followed by Alexa Fluor 488-conjugated donkey anti-goat IgG (1:250, ab150129, Abcam) and Alexa Fluor 594-conjugated donkey anti-rabbit IgG H&L (1:250, ab150076, Abcam) for 1 h at room temperature. Sections were further incubated with DAPI to stain nuclei and observed under a microscope. Immunohistochemical staining of PPARγ (1:100, MBS8241690, Mybiosource) was performed as previously described (23). Adipocyte sizes were determined using Image J, as described previously (24).

### Statistical Analysis

The results were presented as the mean ± standard deviation. Statistical evaluation was performed *via* a nonparametric two-tailed Student’s *t*-test using GraphPad Prism version 6 (www.graphpad.com). Bonferroni’s correction for multiple testing was used for **Figures 4** and **5**. P < 0.05 was considered significant.

## Results

### A Lineage-Tracing System to Label Endosteal Osteoblasts

**Figure 2:**
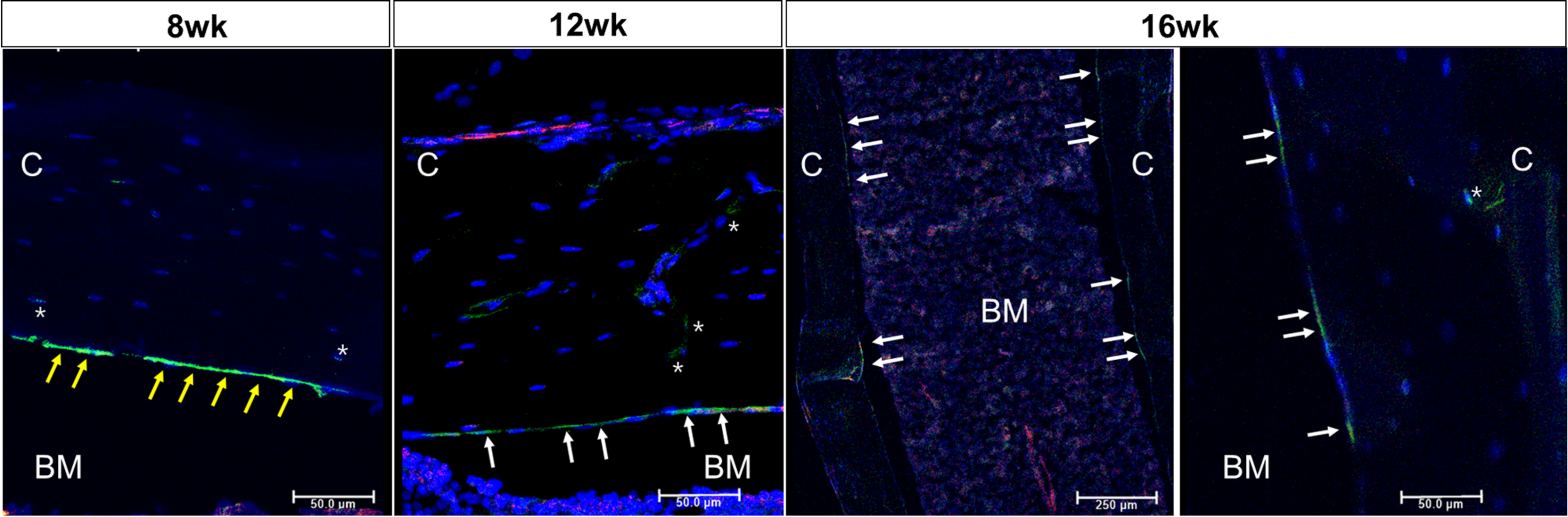
Osteoblast lineage cells are labeled in 10-kb Dmp1- CreERt2:mTmG mice Representative confocal microscopy images of endocortical surface of the femur from Dmp1- CreERt2:mTmG mice at the indicated experimental groups(the left panel at 16 weeks shows low power field). BM, bone marrow; C, cortical bone; white arrow, bone lining cell; yellow arrow, active osteoblast; asterisk, osteocyte; red, mTomato; green, mGFP; blue, DAPI staining of nuclei. Red and green protein fluorescence were captured directly. Scale bar: 50 µm.

**Figure 3:**
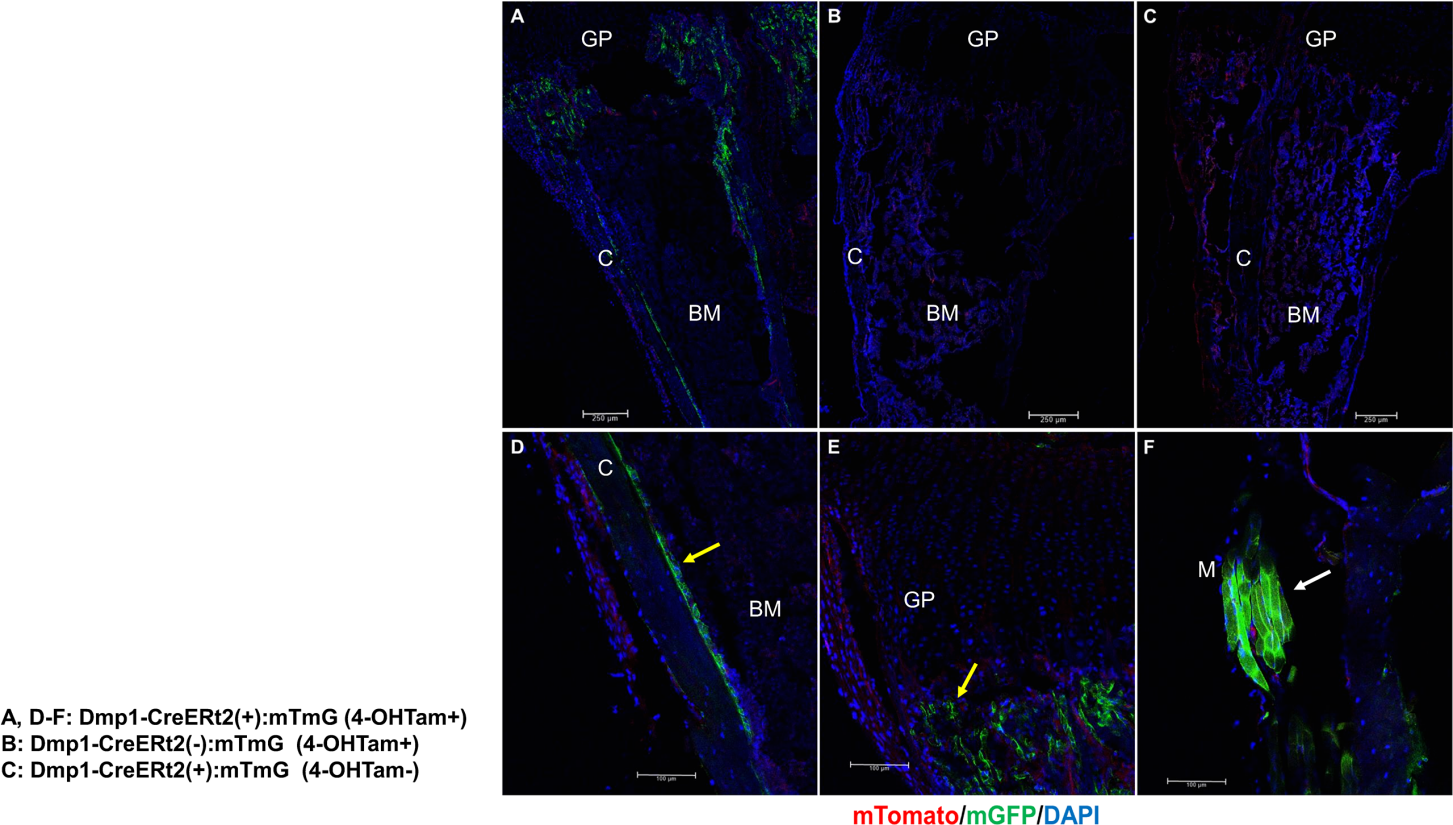
10-kb Dmp1- CreERt2 targets osteoblast lineage cells and skeletal muscle, but not bone marrow cells in 2-week-old mice. **(A–C)** Confocal microscopy images of direct fluorescence on longitudinal sections of the tibia at the indicated experimental groups. **(D–F)** Images at a higher magnification for different areas of bone as indicated. C, cortical bone; GP, growth plate; M, skeletal muscle; yellow arrow, osteoblast; white arrow, muscle; red, mTomato; green, mGFP; blue, DAPI staining of nuclei. Red and green protein fluorescence were captured directly.

**Figure 1** outlines the protocol used for this experiment. As found in previous studies, large numbers of GFP+ (direct fluorescence from mG) plump osteoblasts (yellow arrow) were identified on the endosteal surfaces of the femur 2 days after the last 4-OHTam injection in postnatal week 8 (**Figure 2**). Four weeks after the last 4-OHTam injection (12-week-old mice), the GFP+ cells, presumably BLCs (white arrow), became flat and less numerous on the endosteal surface of the femur. A few GFP+ osteocytes are shown in **Figure 2** (asterisk). The flat GFP+ cells persisted on the endosteal surface in mice at week 16. Therefore, we could label mature osteoblasts and BLCs in the 10-kb Dmp1-CreERt2:mTmG mice. Additionally, to characterize the targeting specificity of 10-kb Dmp1-CreERt2:mTmG, we analyzed GFP expression in sections of the tibia at 2 weeks of age (**Figure 3**). The Dmp1-CreERt2:mTmG mice showed strong GFP signals in the tibia but not in the BM (**Figure 3A**). As expected, the tibia sections from CreERt2(-):mTmG mice did not exhibit any GFP activity (**Figure 3B**). Furthermore, GFP+ cells were not detected on the bone sections of Dmp1-CreERT2:mTmG mice without 4-OHTam(**Figure 3C**). Within the tibia, Dmp1-CreERt2 marked not only endocortical osteoblasts (**Figure 3D**) but also trabecular osteoblasts (**Figure 3E**) and the adjacent skeletal muscle (**Figure 3F**).

### Scl-Ab Reverses the Negative Effects of Rosiglitazone on Bone Parameters

**Figure 4:**
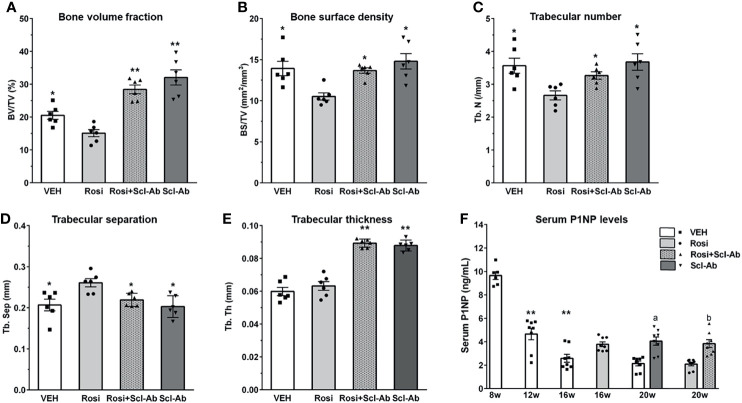
Scl-Ab rescues the negative effects of rosiglitazone on bone parameters. **(A–E)** MicroCT quantification of trabecular bone. Each data represents the mean, and error bars represent the standard error (*, P < 0.05 vs. rosiglitazone; **, P < 0.01 vs. rosiglitazone, N = 6/group). BV, bone volume; TV, total volume; BS, bone surface, Tb.N, trabeculae number; Tb.Sp, trabeculae separation; Tb.Th, trabeculae thickness. **(F)** Serum P1NP measurements from mice in the indicated groups. Each data represents the mean, and error bars represent the standard error (**, P < 0.01 vs. 8 week; a, P < 0.05 vs. vehicle; b, P < 0.05 vs. rosiglitazone, N = 6–8/group). Vehicle, white; rosiglitazone, gray; Scl-Ab, dark gray; Scl-Ab/rosiglitazone, gray pattern.

We first analyzed the skeletal effects of 8 weeks of exposure to rosiglitazone by measuring microCT parameters of the distal femur. Significant decreases in BV/TV (**Figure 4A**), bone surface density (BS/TV) (**Figure 4B**), and Tb.N (**Figure 4C**), and increases in Tb.Sp (**Figure 4D**) were noted after 8 weeks of rosiglitazone administration compared to the effects of the vehicle (P < 0.05). We then examined the effects of Scl-Ab in rosiglitazone-treated and control mice. Scl-Ab administration significantly improved trabecular parameters induced by rosiglitazone (BV/TV and Tb.Th: P < 0.01; BS/TV, Tb.N, Tb.Sp, and serum P1NP levels: P < 0.05) (**Figure 4**). The combined use of rosiglitazone and Scl-Ab did not lead to significant differences in these parameters compared to the effects of Scl-Ab alone. Serum P1NP levels significantly decreased from 8 to 12 weeks (P < 0.01). Scl-Ab significantly increased serum P1NP levels versus the vehicle (P < 0.05). In addition, 4 weeks of overlapping Scl-Ab treatment significantly increased serum P1NP levels compared to the effects of rosiglitazone alone (P < 0.05, **Figure 4F**).

### Rosiglitazone Increases GFP/Perilipin Double+ BMAds, Whereas Scl-Ab Modestly Suppresses This Effect

**Figure 5:**
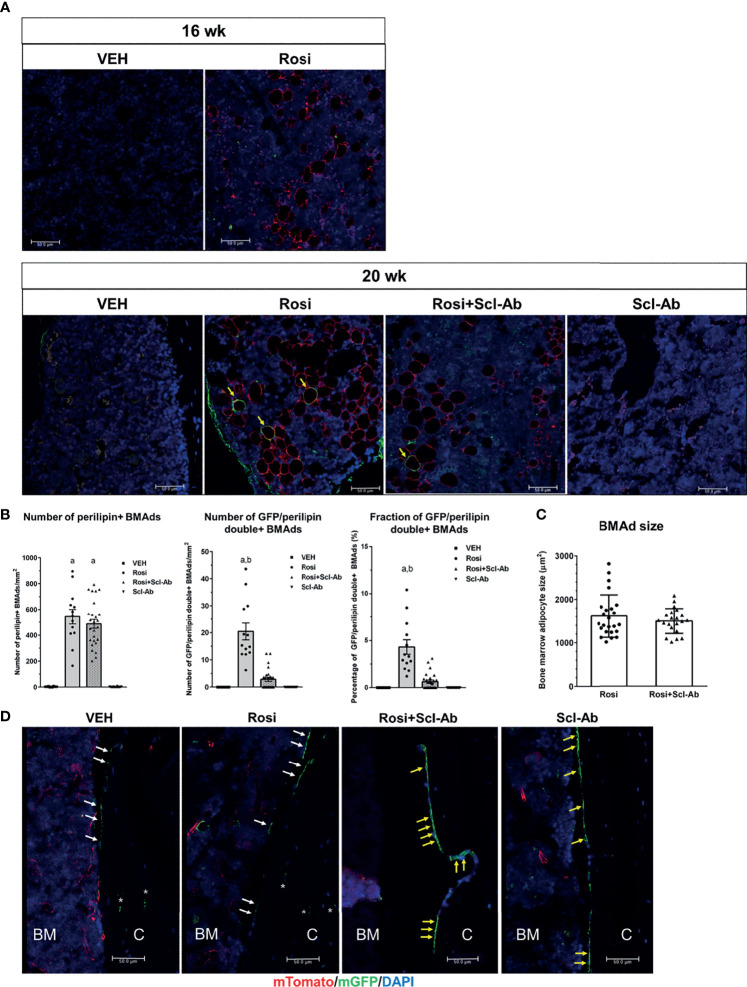
Rosiglitazone increases GFP/perilipin double positive bone marrow adipocytes, whereas Scl-Ab suppresses the effect of rosiglitazone on them. **(A)** Representative immunostaining against GFP and perilipin on sections of femoral bone marrow from Dmp1- CreERt2:mTmG mice in the indicated experimental groups. Scale bar: 50 µm. Red, perilipin+ bone marrow adipocytes (BMAds); yellow arrow, GFP/perilipin double positive BMAds; blue, DAPI staining of nuclei. **(B)** Quantification of total perilipin+ or GFP/perilipin double+ BMAds. a, P < 0.001 vs. vehicle or Scl-Ab; b, P < 0.001 vs. rosiglitazone + Scl-Ab; N=4–6/group. **(C)** Quantification of perilipin+ BMAd size in the indicated experimental groups (N = 4–6/group). **(D)** Representative confocal images showing direct fluorescence in cells on endocortical surfaces of the femur from the indicated experimental groups. BM, bone marrow; C, cortical bone; white arrow, bone lining cell; yellow arrow, active osteoblast; asterisk, osteocyte; red, mTomato; green, mGFP; blue, DAPI staining of nuclei. Red and green protein fluorescence were captured directly. Scale bar: 50 µm.

To determine whether rosiglitazone could stimulate the differentiation of osteoblast lineage cells into BMAds, we chased GFP+ cells on the endosteal surface of the femur after 8 weeks of rosiglitazone administration. To better characterize and quantify the number of BMAds, we performed immunohistochemical staining using anti-perilipin antibody and anti-GFP antibody. Therefore, BMAds were visualized with anti-perilipin antibody, and GFP/perilipin double+ BMAds should be derived from osteoblast lineage cells. Representative histological images of BMAds in the femur in each group are shown in **Figure 5A**. After a 4-week chase period (16 weeks), some perilipin+ BMAds were detected in the BM but no GFP/perilipin double+ BMAds were detected. After an 8-week chase period, GFP/perilipin dual expression was detected from a few BMAds (yellow arrow) in the rosiglitazone group, and very few GFP/perilipin double+ BMAds were detected in the rosiglitazone/Scl-Ab group (N = 4–6/group). As shown in **Figure 5B**, rosiglitazone significantly increased the overall number of adipocytes (perilipin + BMAds) in the femur BM (rosiglitazone 543.5 ± 205.9/mm^2^ vs. vehicle 3.1 ± 2.1/mm^2^; P < 0.001, **Figure 5A**). Moreover, some GFP/perilipin double+ BMAds were identified near the endosteal surface (rosiglitazone 20.5 ± 11.2/mm^2^ vs. vehicle 0/mm^2^; P < 0.001, **Figure 5B**). Collectively, these data suggest that a small subpopulation of endosteal osteoblast lineage cells could differentiate into BMAds during rosiglitazone treatment compared to the vehicle findings (rosiglitazone 4.3 ± 2.8% vs. vehicle 0%; P < 0.001, **Figure 5B**).

We then tested whether Scl-Ab could modulate this effect of rosiglitazone on BMAds and thereby alter the total number of BMAds and the number of GFP/perilipin double+ BMAds in rosiglitazone-treated mice. When 8 weeks of rosiglitazone was overlapped by 4 weeks of Scl-Ab treatment, the total number of BMAds was not significantly decreased compared to the effects of 4 weeks of vehicle treatment (rosiglitazone 543.5 ± 205.9/mm^2^ vs. Scl-Ab/rosiglitazone 487.3 ± 175.2/mm^2^, **Figure 5B**). Additionally, Scl-Ab did not decrease the adipocyte size significantly (rosiglitazone 1612.2 ± 490.1 µm^2^ vs. Scl-Ab/rosiglitazone 1498.5 ± 286.6 µm^2^, **Figure 5C**). Interestingly, Scl-Ab significantly decreased the number of GFP/perilipin double+ BMAds in the femur BM relative to the vehicle (rosiglitazone 20.5 ± 11.2/mm^2^ vs. Scl-Ab/rosiglitazone 2.8 ± 3.7/mm^2^; P < 0.001, **Figure 5B**). Collectively, when we quantified the contribution of the differentiation of osteoblast lineage cells into adipocytes to the entire BM adipogenesis process by counting the number of GFP/perilipin double+ BMAds and total perilipin+ BMAds, Scl-Ab preferentially suppressed the differentiation of osteoblast lineage cells into adipocytes (rosiglitazone 4.3 ± 2.8% vs. Scl-Ab/rosiglitazone 0.6 ± 0.9%; P < 0.001, **Figure 5B**). In addition, as found in previous studies, more GFP+ endosteal cells appeared plumper in the Scl-Ab or Rosi+Scl-Ab treatment groups than in the vehicle or Rosi alone groups (**Figure 5D**).

**Figure 6:**
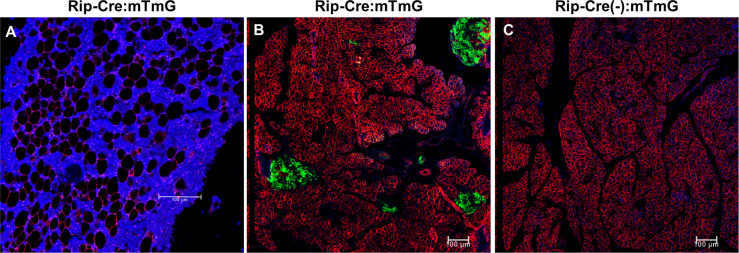
Rosiglitazone does not increase endogenous GFP expression. **(A)** Representative immunostaining against GFP and perilipin on sections of femoral bone marrow from Rip-Cre:mTmG mice that had received rosiglitazone for 8 weeks. Red, perilipin+ bone marrow adipocytes; blue, DAPI staining of nuclei. **(B, C)** Representative confocal images of the pancreas from Rip-Cre:mTmG mice or Rip-Cre(-):mTmG mice that had received rosiglitazone for 8 weeks. Red, mTomato; green, mGFP; blue, DAPI staining of nuclei. Rip-Cre transgene, a 668 bp fragment of the rat insulin II promoter. Red and green protein fluorescence were captured directly. Scale bar: 100 µm.

To exclude the possibility that rosiglitazone affected endogenous GFP expression, Rip-Cre:mTmG mice were also treated with rosiglitazone. In Rip-Cre transgenic mice, the rat insulin II promoter drives cre expression in both β cells in the pancreas and some neurons in the brain (25). Co-immunostaining experiments showed that rosiglitazone also increased the number of perilipin+BMAds in Rip-Cre:mTmG mice, but GFP/perilipin double+ cells were not detected on the endosteal surface or in the BM of the femur (**Figure 6A**). As expected, Rip-Cre mice targeted islet cells in the pancreas (**Figure 6B**), and no GFP signals were detected in the pancreas sections obtained from Rip-Cre(-):mTmG mice (**Figure 6C**). Thus, rosiglitazone did not increase endogenous GFP expression.

### Osteoblasts and BLCs Express PPARγ

**Figure 7:**
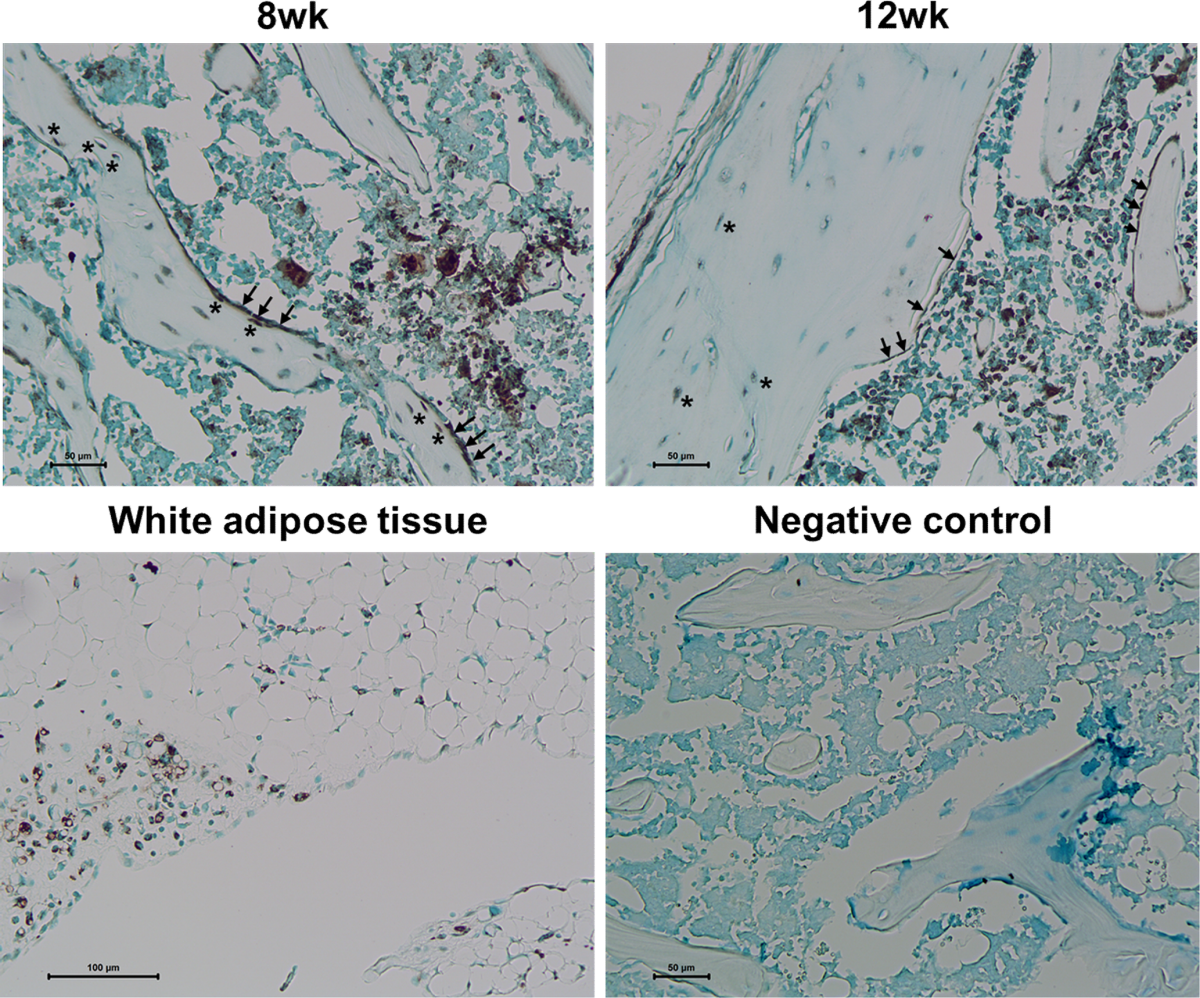
PPARγ expression in osteoblasts and bone lining cells. Representative images for PPARγ immunohistochemical staining of osteoblast lineage cells (arrow) and osteocytes (asterisk) in the femur at 8 and 12 weeks. Immunostaining is shown as a brown color in white adipose tissue. Staining was absent in a negative control section. The primary antibody was replaced with nonimmune serum (1: 100) for negative control slides. Tissues were counterstained with methyl green (N = 3/each group).

To explore the adipogenic characteristics of osteoblast lineage cells in response to rosiglitazone treatment, we examined PPARγ expression. Immunohistochemical staining revealed numerous PPARγ−expressing cells in the trabecular and cortical bones, which were likely to be osteoblasts and BLCs in the femur at 8 and 12 weeks, respectively (**Figure 7**).

## Discussion

In this study, we demonstrated that rosiglitazone could stimulate the transdifferentiation of endosteal osteoblast lineage cells such as BLCs into BMAds, which appeared to contribute to increased BMA. In addition, overlapping Scl-Ab administration could suppress this effect. To the best of our knowledge, this is the first report to reveal that BLCs serve as sources of BMAds using a lineage-tracing study.

In the present study, rosiglitazone increased the number of BMAds and concurrently decreased bone mass. These data further strengthened the association between BMA and bone mass. In humans, pioglitazone, another PPARγ agonist, has also been demonstrated to influence both BMA and bone mass (26). A previous 12-month study recorded a statistically significant correlation between the increase in femoral BMA and decrease in hip BMD (26). Although the association between BMA and BMD appears clear, the mechanism of action has not been clarified. One likely possibility is that intrinsic and extrinsic signals determine mesenchymal stem cell (MSC) lineage differentiation into osteoblasts or adipocytes (27–29). Most rosiglitazone-induced BMAds are proposed to be derived from MSCs. Although the molecular mechanisms by which MSCs differentiate to adipocytes remain unclear, multiple transcription factors have been demonstrated to be critical for the differentiation of MSCs into adipocytes. PPARγ and C/EBPs are involved in the adipogenic differentiation of MSCs (30–33). Alternatively, the dedifferentiation or transdifferentiation of osteoblast lineage cells could be a potential route to BMAds (20). In this study, we observed that a small subpopulation of osteoblast lineage cells has the capacity to differentiate into BMAds. However, this result does not completely exclude the possibility that mature osteoblasts or osteocytes directly differentiate into BMAds. In addition, we could not detect cells that have characteristics of both osteoblasts and adipocytes undergoing the transition from BLCs to BMAds in the BM. Although it remains unclear that BLCs directly transdifferentiate into BMAds, BLCs could be a promising source of BMAds. In previous lineage tracing studies, we demonstrated that Dmp1-CreERt2 or osteocalcin-CreERt2 mature osteoblasts differentiate into BLCs *in vivo* (17, 18). Mature osteoblasts could become inactive in the first step so that they transdifferentiate into BMAds. Additionally, osteocyte dedifferentiation into osteoblasts or BLCs could be possible, but this has not been reported to occur in physiologic *in vivo* settings. Collectively, mature osteoblasts and their progeny could be a source of BMAds.

The mechanism by which osteoblast lineage cells differentiate into BMAds is unclear. In a previous study, we found that PPARγ-overexpressed preosteoblasts can transdifferentiate into adipocyte-like cells *in vitro (*34). Furthermore, we demonstrated that PPARγ activation inhibits osteocalcin expression both by suppressing Runx2 expression and interfering with the transactivation of Runx2 in osteoblast cell lines (35). In this study, we found that even endosteal lining cells can express PPARγ. BLCs could express cell-surface markers characteristic of mesenchymal stem/progenitors (19). Collectively, some subpopulations of osteoblast lineage cells containing MSC-like characteristics or expressing PPARγ might respond to external signals that stimulate differentiation into adipocytes.

We found that Scl-Ab significantly suppressed the transdifferentiation of osteoblast lineage cells into BMAds. The anabolic action of Scl-Ab is predominantly modeling-based bone formation (36). One primary cellular mechanism of modeling-based bone formation is the reactivation of inactive BLCs into active osteoblasts (17). The mechanism by which this suppressive effect of Scl-Ab on the adipogenic differentiation of osteoblast lineage cells could contribute to anabolic action is unclear. The conditional deletion of Sost in Prx1-expressing cells recapitulated the global high-bone-mass phenotype of Sost KO mice, whereas both Col1- and Dmp1-specific deletions of Sost induced milder increases in bone formation (37). Thus, although cell populations of osteoblasts at various differentiation stages could be targets of Scl-Ab, MSCs or mesenchymal progenitor cells appear to be the main targets.

Few studies have revealed a link between sclerostin and the regulation of BMAT development. Recombinant Sost or Sost in osteocyte-conditioned medium stimulated BM adipogenesis (38). Furthermore, Scl-Ab treatment reversed the increase in marrow adiposity in ovariectomized rabbits (39). A recent lineage tracing study showed that Scl-Ab administration suppresses adipogenesis by suppressing the differentiation of Sox9+ skeletal precursors into BMAds *in vivo* (40).

Several questions remain to be addressed. Overlap of 4 week-Scl-Ab administration failed to inhibit the increase in BMAd counts of the femur induced by 8 weeks of rosiglitazone administration, whereas the negative effect on bone was significantly improved. However, a recent study revealed that Scl-Ab significantly decreased rosiglitazone-induced BMAT in the femur (41). The discrepancy could have resulted from differences of the doses or administration schedules of Scl-Ab. In the current study, we focused on the possibility that BLCs could transdifferentiate into BMAds. Thus, we adopted distinct administration schedules of rosiglitazone and Scl-Ab from other studies. Another possibility of the discrepancy is that osteoblasts lineage cells could have distinct responses to rosiglitazone in the adipogenic differentiation from that of MSCs. In this study, after a 4-week chase period (16 weeks), some perilipin+ BMAds were detected in the BM but there were no GFP/perilipin double+ BMAds. Therefore, osteoblast lineage cell differentiation into BMAds in response to rosiglitazone could require more time compared to MSCs. Furthermore, we did not use osmium tetroxide micro-CT analysis to evaluate adipose tissue volume. Next, in this experiment, we focused on presumably regulated BMAT (rBMAT) in the femur because it might be more dynamic than constitutive BMAT (cBMAT). Previous studies revealed the presence of cBMAT and rBMAT as distinct BMAT subpopulations in different periods of development in rodents (42). rBMAT has been proposed to be preferentially distributed within the mid-to-proximal tibia, femur, and lumbar vertebrae, whereas cBMAT develops in the distal tibia and caudal vertebrae from an early stage (11). However, further studies are required to determine whether cBMAT and rBMAT are differentially regulated by Sost. In addition, there was an issue of targeting of Dmp1-CreERt2. We could not detect GFP+ cells in the BM of Dmp1-CreERt2:mT/mG mice at 8 weeks, 12 weeks, and 16 weeks. Additionally, there were no GFP+ cells in the BM of RIP-Cre:mTmG mouse treated with rosiglitazone for 8 weeks. However, this do not completely exclude ectopic expression of the current 10kb Dmp1-CreERt2 in BM. Previous studies suggested that the 14kb Dmp1-Cre targets not only osteoblasts and osteocytes but also osteoblast precursors at an earlier stage (43). However, the cre activity was not detected in BMAds in that study. Therefore, it is most unlikely that the 10kb Dmp1-CreERt2 exists in BMAd precursors.

Moreover, we could not identify the genetic characteristics of osteoblast lineage cells that could differentiate to BMAds. It would be more valuable to compare the genetic characteristics of osteoblast-derived and MSC-derived adipocytes. Finally, we observed weak immunostaining of PPARγ in osteoblast lineage cells. More sophisticated experiments are necessary to confirm this result.

In conclusion, BLCs could represent sources of BMAds, and the pharmacologic inhibition of sclerostin might suppress the differentiation of osteoblast lineage cells into BMAds in response to BM adipogenic signals.

## Data Availability Statement

The original contributions presented in the study are included in the article/supplementary material. Further inquiries can be directed to the corresponding author.

## Ethics Statement

The animal study was reviewed and approved by the Institutional Animal Care and Use Committee of Seoul National University.

## Author Contributions

All authors designed experiments and interpreted experiments. JL and JY performed experiments. SK wrote the manuscript. All authors contributed to the article and approved the submitted version.

## Funding

This work was supported by grants from the National Research Foundation of Korea (2017R1A2B2004708).

## Conflict of Interest

The authors declare that the research was conducted in the absence of any commercial or financial relationships that could be construed as a potential conflict of interest.

## Publisher’s Note

All claims expressed in this article are solely those of the authors and do not necessarily represent those of their affiliated organizations, or those of the publisher, the editors and the reviewers. Any product that may be evaluated in this article, or claim that may be made by its manufacturer, is not guaranteed or endorsed by the publisher.

## Abbreviations

BM, Bone marrow; BMA, Bone marrow adiposity; BMAd, Bone marrow adipocyte; BMAT, Bone marrow adipose Tissue; BMD, Bone mineral density; BV/TV, Bone volume/total volume; BS/TV, Bone surface/total volume; Tb. Th, Trabecular thickness; Tb. N, Trabecular number; Tb. Sp, Trabecular separation.
